# Characterization of PTZ-Induced Seizure Susceptibility in a Down Syndrome Mouse Model That Overexpresses *CSTB*

**DOI:** 10.1371/journal.pone.0027845

**Published:** 2011-11-30

**Authors:** Véronique Brault, Benoît Martin, Nathalie Costet, Jean-Charles Bizot, Yann Hérault

**Affiliations:** 1 Department of Translational Medicine and Neurogenetics, Institut de Génétique Biologie Moléculaire et Cellulaire (IGBMC), Inserm U596, CNRS UMR7104, Université de Strasbourg, Illkirch, France; 2 Inserm U642, Rennes, France; 3 Laboratoire Traitement du Signal et de l'Image, Université de Rennes 1, Rennes, France; 4 Key-Obs S.A.S., Orléans, France; 5 Transgenese et Archivage Animaux Modèles, TAAM, CNRS, UPS44, Orléans, France; 6 Institut Français Clinique de la Souris, GIE CERBM, Illkirch, France; Institut Jacques Monod, France

## Abstract

Down syndrome (DS) is a complex genetic syndrome characterized by intellectual disability, dysmorphism and variable additional physiological traits. Current research progress has begun to decipher the neural mechanisms underlying cognitive impairment, leading to new therapeutic perspectives. Pentylenetetrazol (PTZ) has recently been found to have positive effects on learning and memory capacities of a DS mouse model and is foreseen to treat DS patients. But PTZ is also known to be a convulsant drug at higher dose and DS persons are more prone to epileptic seizures than the general population. This raises concerns over what long-term effects of treatment might be in the DS population. The cause of increased propensity for epilepsy in the DS population and which Hsa21 gene(s) are implicated remain unknown. Among Hsa21 candidate genes in epilepsy, *CSTB*, coding for the cystein protease inhibitor cystatin B, is involved in progressive myoclonus epilepsy and ataxia in both mice and human. Thus we aim to evaluate the effect of an increase in *Cstb* gene dosage on spontaneous epileptic activity and susceptibility to PTZ-induced seizure. To this end we generated a new mouse model trisomic for *Cstb* by homologous recombination. We verified that increasing copy number of *Cstb* from Trisomy (Ts) to Tetrasomy (Tt) was driving overexpression of the gene in the brain, we checked transgenic animals for presence of locomotor activity and electroencephalogram (EEG) abnormalities characteristic of myoclonic epilepsy and we tested if those animals were prone to PTZ-induced seizure. Overall, the results of the analysis shows that an increase in *Cstb* does not induce any spontaneous epileptic activity and neither increase or decrease the propensity of Ts and Tt mice to myoclonic seizures suggesting that *Ctsb* dosage should not interfere with PTZ-treatment.

## Introduction

Down syndrome, resulting from the presence of an extra copy of human chromosome 21 (Hsa21 for Homo sapiens chromosome 21), is the major genetic cause of cognitive disabilities [1]. DS is associated with learning and memory defects, implicating dysfunction of hippocampal pathways [2]–[5]. The mouse Ts65Dn model, trisomic for a segment of mouse chromosome (Mmu for Mus musculus) 16 containing approximately 60% of the Hsa21 orthologous genes [6], has been shown to mimic DS deficits in learning and memory [7], [8]. Ts65Dn mice were used to test several therapeutic interventions to improve learning and memory [9]. Two independent studies successfully administrated chronic low dose of the GABA_A_ antagonist PTZ to restore LTP and cognition in Ts65Dn mice [10], [11]. PTZ is also known for its ability at higher dose to induce seizure, by impairing GABA-mediated inhibition [12], [13]. Frequency of epilepsy in DS has been reported ranging from 6 to 17% [14]–[16] with phenotype features varying with the age of the patient and a triphasic distribution of seizure onset (infancy, early adulthood and late onset) having been suggested [17]. A prevalence reaching 46% in patients over 50 years was even reported [14], [16]. Thus using PTZ to treat DS people raises concerns about potential long-term side effects.

An interesting candidate for susceptibility to epilepsy in DS is *CSTB*, a gene located on Hsa21 and shown to be overexpressed in the brain of DS individuals [18], but whose mouse ortholog is absent in the Ts65Dn model used to test PTZ treatment. Mutations in *CSTB* are associated with progressive myoclonus epilepsies (PMEs) in Unverricht-Lundborg disease (EPM1; OMIM254800) [19]–[21] a disease that shares features with late myoclonic epilepsy observed in DS [22]. At least 10 isoforms of CSTB have been reported with pathologic influence, leading to EPM1. In 90% of the cases, EPM1 results from a down regulation of gene expression due to the expansion of a dodecamer repeat in the putative promoter of CSTB [19]–[21], with a polymorphism of 2 or 3 copies existing in individuals without EPM1 [23] and asymptomatic pre-mutation alleles of 12–17 repeats leading to reduced mRNA levels [24]. As expected *Cstb* loss-of-function induces EPM1-like phenotypes in the mouse [25] and it has been postulated that *CSTB* deficiency increases susceptibility to generalized tonico-clonic seizures and seizure-induced cell death [26]. Reduced density of GABA-immunoreactive cells in the hippocampus of *Cstb*-deficient mice and increased susceptibility to kainate-induced seizures of those mice suggest a defect of the GABAergic system. If increased synthesis of CSTB after induced seizures has been suggested to have an anti-apoptotic role [27], it is not known what effect might have a persistent increased amount of cystatin B on the cell. While human cystatin C, another protein from the same family, is a well known amyloid protein involved in human cerebral amyloid angiopathy [28], cystatin B was shown to interact with amyloid-beta peptide of Alzheimer's disease both in vitro and in the cells and can form aggregates in cells [29], [30]. Therefore increased Cstb might, as well as its absence, have a deleterious effect on the cell.

In the present study, we tested two related hypothesis regarding a potential role of *Cstb* in the pathogenesis of epilepsy: (1) Overexpression of *Cstb*, like underexpression, could induce EPM1-like phenotypes; and (2) *Cstb* could be a candidate gene for increased susceptibility to epilepsy in DS. We took advantage of a genetically engineered transgenic line carrying a tandem duplication of *Cstb* to test if change in *Cstb* dosage could induce a spontaneous epileptic activity or modify PTZ-induced seizure susceptibility. After verifying the increased gene expression in heterozygous (trisomic-Ts) and homozygous (tetrasomic-Tt) mice, we tested those mice for locomotor and electrophysiological brain activities, and propensity to clonic seizures after PTZ administration.

## Results

### Creation of transgenic mice trisomic and tetrasomic for *Cstb* by generating a tandem duplication of *Cstb*

**Figure 1:**
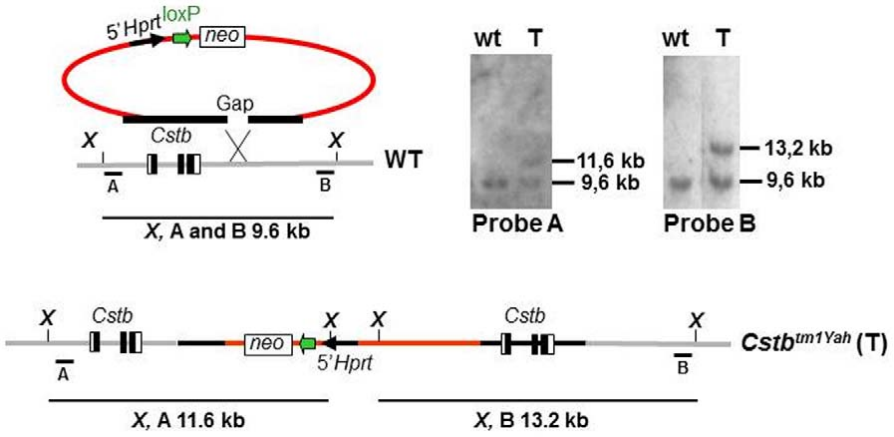
Generation of a tandem duplication of the *Cstb* gene on Mmu10. The targeting vector containing a loxP site (green arrow), a selectable antibiotic resistance gene (neo), and the 5′part of the *Hprt* gene were integrated in the *Cstb* locus (*Cstbtm1Yah*), leading to the tandem duplication of *Cstb*. The *Cstb<tm1Yah>* allele was checked by Southern analysis with probes A and B, and BstXI restriction enzyme, showing a fragment of 9.6 kb for the wild-type allele (wt) and a 11.6 kb fragment (probe A) or a 13.2 kb fragment (probe B) for the *Cstb<tm1Yah>* (T) allele.

The tandem duplication of *Cstb* on MMU10 was generated *in vivo* by chromosomal engineering [31]. The vector containing the genomic sequence (MMU10 229945–237205 in NCBIm37 mouse assembly) that bears the *Cstb* allele was selected from the 5′*Hprt* library [32]. It was integrated into the *Cstb* locus by targeting in HM-1 ES cells [33] and confirmed by different restriction enzymes and specific probes (Fig. 1). We derived a *Cstb<tm1Yah>* mouse line from the ES with the integrated vector (T). Mosaic animals were bred with B6 mice to establish a heterozygous (trisomic) line. Afterward, Ts animals were intercrossed to produce 2n, Ts and Tt mice for experimental analyses. We observed a normal Mendelian segregation ratio for both the heterozygous and homozygous animals. Some animals reached the age of 6 month without any apparent pathology developing. Hence, the transgenic animals were all viable, fertile and healthy.

### Increased *Cstb* expression in the liver and brain of *Cstb* transgenic mice

**Figure 2:**
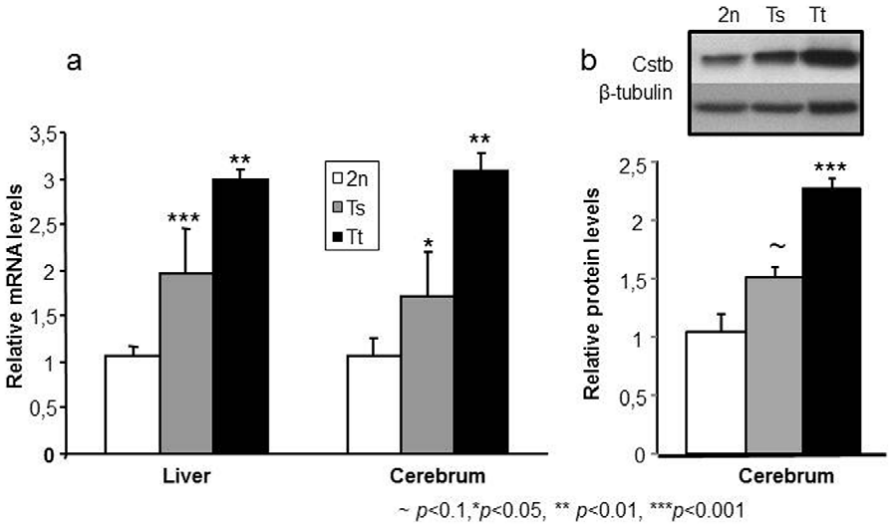
Analysis of *Cstb* expression in the liver and cerebrum of mice that are disomic (2n), trisomic (Ts) and tetrasomic (Tt) for *Cstb*. (a) Real-time PCR analysis: mRNA levels are expressed relative to the disomique control. Data are represented as mean±sem. In both liver and cerebrum, *Cstb* expression is increased by ∼2 folds in the Ts and by ∼3 folds in the Tt mice. (b) Western blot analysis: band intensities were estimated using ImageJ and normalized against the loading control β-tubulin. Protein levels are represented as fold-changes relative to the 2n control and represented as mean±sem. Amounts of cystatin B are increased by about 1.5 fold in Ts and by about 2.3 fold in Tt brains. Inset shows one representative band for 2n, Ts and Tt.

We verified that the presence of additional copies of *Cstb* resulted in increased expression of the gene in the transgenic mice. For this, we measured *Cstb* mRNA in the liver and brain of 2n, Ts and Tt mice. Levels of transcript expression were assessed in total RNA extracts from livers (8 2n; 8 Ts; 6 Tt) and brains (8 2n; 8 Ts; 7 Tt) by quantitative real-time PCR (QRT-PCR) and relative amount of transcripts were represented with one wild-type sample taken as the reference amount (equal 1). In both liver and brain, *Cstb* expression was increased by about two folds (fold change 1.965, U-test *p*<0.001 for the liver; fold change 1.72 U-test *p* = 0.021 for the brain) in the trisomic mice and by about 3 folds in the tetrasomic ones (fold change 2.994, U-test *p*<0.001 for the liver; fold change 3.08, U-test *p*<0.001 for the brain) (Fig. 2). Hence, the presence of additional copies of *Cstb* results in over expression of the gene. However, increased expressions in trisomic and tetrasomic mice were higher than expected from the number of added copies of the gene (1.5 fold for one additional copy and 2 fold for 2 additional copies). We further investigated if the increased transcript expression also resulted in an increased amount of protein. Amount of Cystatin B was assessed in the brain of 2n (4 animals), Ts (3 animals) and Tt (4 animals) using Western blot analysis. Quantity of protein was normalized to the amount of β-tubulin and relative protein levels in each animal were calculated with one 2n animal as the reference amount (equal 1). Increased in protein levels were observed in (Ts) (fold change 1.5, Student's t-test, *p* = 0,067) and Tt (fold change 2.2, Student's t-test, *p* = 0.0004) mice, but was slightly lower that the increase observed at the mRNA level.

### Looking for signs of ataxia, cerebellar atrophy and epilepsy in Ts and Tt mice: locomotion, histological analysis and electrophysiological recordings

**Figure 3:**
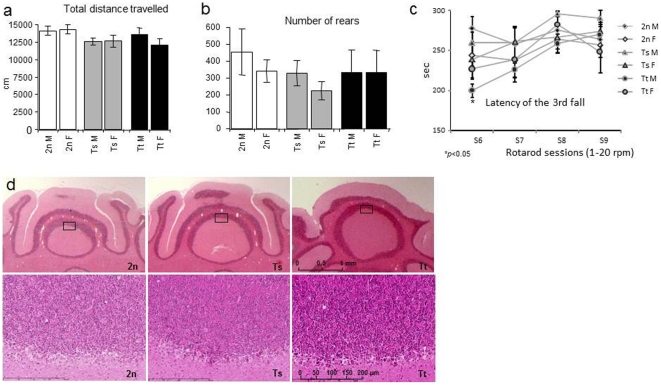
Locomotor activity of 2n, Ts and Tt mice and histological analysis of the cerebellum. (a) and (b) Mean ± SEM for the total distance travelled and the number of rears during the 30 min session in the open field. (c) Mean ± SEM for the latency to fall from the ratorod. (d) Hematoxylin/eosin-stained coronal sections in through the cerebellum of 6 month-old 2n, Ts and Tt mice (×1,25 and ×40 magnifications) showing similar granular cell layers for the different mice. The position of the enlarged zone in the higher magnifications is shown by the boxes in the top panel.

Knowing that *Cstb* is implicated in progressive ataxia and myoclonic epilepsy, we aimed to check if an excess of cystatin B could trigger the same type of pathologies. Mice were tested for spontaneous locomotor activity in an open-field, for skilled behaviour in rotarod and for spontaneous epileptic cortical activity. Fifteen 2n, ten Ts and eight Tt mice about 6 month of age were tested. Locomotor activity was scored by measuring the travelled distance and the number of rears for a period of 30 min (Fig. 3a, b). Ataxia is characterized by wide-base gait walking with occasional falling upon hindlimb rearing. There was no sign of such atypical behaviour observed and no difference in the distance travelled and the number of rears between the different groups of mice. The 3^rd^ fall latency was not significantly different between groups on the first five sessions of rotarod (low speed: 1–5 rpm; data not shown) and was significantly different between groups on sixth session (ANOVA: *p* = 0.03), but not on the following sessions (high speed: 1–20 rpm; Fig. 3c). Post-hoc comparison by the Student's t-test shows that the 3^rd^ fall latency was lower in Tt male mice than in 2n male mice on the 6^th^ session (S6 in Fig. 3c; *p* = 0.02); the other post-hoc comparisons did not reveal significant difference. We compared gross histology of cerebellar tissues from 2n, Ts and Tt mice aged between 6 and 10 months. The cerebellum of *Cstb* knock-out mice characterized by a dramatic shrank evident at the macroscopic level and by a drastic reduction of the cell density of the granule cell layer visible at low-resolution analysis of histological sections [25], [26]. We could see no such sign of cerebellar atrophy (data not shown) and no visible change in the density of the granular cell layer in the cerebellum of in all populations of mice (Fig. 3d).

**Figure 4:**
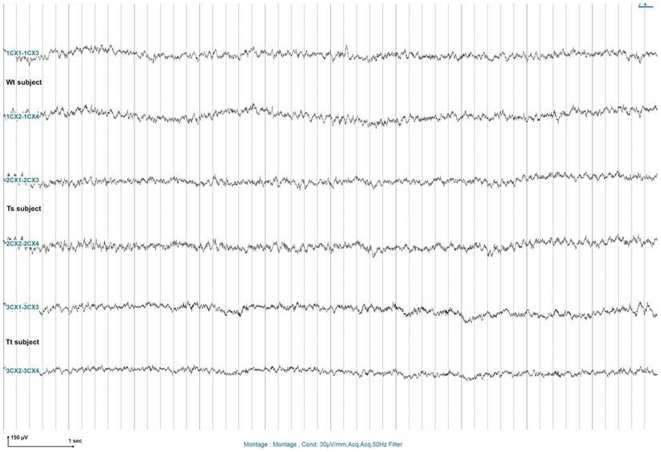
Electrocorticographic activity of 2n, Ts and Tt mice. Trace from left and right hemisphere show normal cortical activity. Calibration: 30 µV/mm.

Spontaneous myoclonic seizures observed in *Cstb*-deficient mice appear already at one month of age and are associated with characteristic EEG [26]. This phenotype was investigated in young 2n, Ts and Tt adult mice. For this, mice were monitored for their cortical activity using EEG recording. No seizures or epileptiform abnormalities were detected in wild-type or in transgenic mice suggesting an absence of any epileptic activity for both populations of mice (Fig. 4).

### Testing susceptibility of *Cstb* trisomic and tetrasomic mice to PTZ-induced seizures

**Figure 5:**
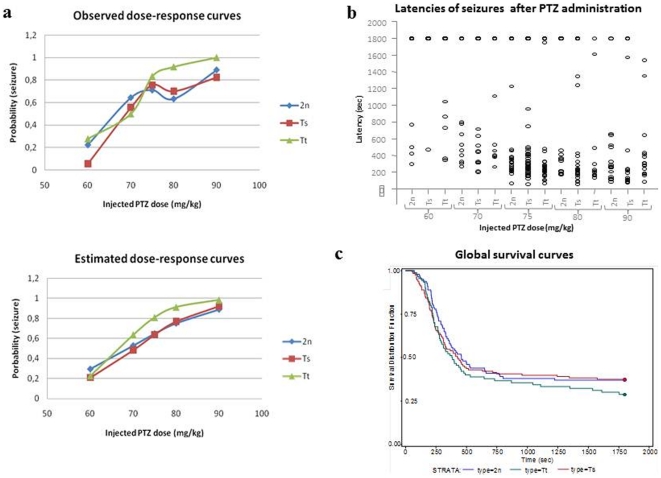
Evaluation of susceptibility of 2n, Ts and Tt mice to PTZ-induced seizure. (a) Dose-response curves showing the ratio of the number of convulsing mice observed (obs) or predicted (pred) to the total number of injected animals for each PTZ dose. (b) Distributions of latencies of seizure for each genotype at the different doses of PTZ administered. (c) Global survival curves of 2n (blue), Ts (red) and Tt (green) mice (probability of seizure according to time, latencies right censored at 1800 sec).

To see if the presence of additional copies of *Cstb* could modify the mouse susceptibility to seizure, we challenged control 2n, Ts and Tt mice to the seizure-provoking agent PTZ. PTZ decreases the potency of GABA-mediated inhibition in brain [34] and, depending on dosage, can produce myoclonic jerks, tonico-clonic convulsions followed or not by tonic seizures in animals. Mice were injected with increasing doses of PTZ and the number of mice showing tonico-clonic seizures was recorded for each genetic group and at different doses. In all three groups of mice, the administration of PTZ induced convulsions in a dose-dependent manner (Table 1 and Fig. 5a). The ED_50_ values of PTZ for Ts and Tt animals were respectively 70.5 [65.5–73.6] and 66.9 [62.0–70.0], and did not differ significantly from the ED_50_ value for control 2n animals, which was 68.9 [60.6–73.4]. The logistic regression model including the genotype of the mouse and the PTZ dose and their interaction to predict the logit of the probability of seizure indicated a statistically significant effect of the PTZ dose (*p*<0.001) but no significant effect of the genotype (*p* = 0.33) or the interaction (*p* = 0.26). No significant odds-ratio was found when comparing Ts and Tt mice to 2n mice (Table 2). Thus increase in expression of the *Cstb* gene in Ts and Tt animals did not enhance the susceptibility to PTZ-induced seizures. Time latency between PTZ administration and the onset of seizure was very variable among each genotype group with few animals with a very late onset of seizure (Fig. 5b). The log-rank test comparing latency curves from the three genotypes (Fig. 5c) indicated no significant differences (*p* = 0.44). Whereas the differences in the mean values among the treatment groups are greater than would be expected by chance, difference among genetic groups within each single PTZ dose was not significant (see Table 3). While a monotonic dose-effect related to clonic seizure latency was observed for 2n and Tt animals, this was not the case for the Ts (Table 3). However, the dose-response curves (Fig. 5a) between genotypes are similar suggesting that this effect is probably an experimental artifact and has no biological mean.

**Table 1:** Analysis of PTZ-induced tonico-clonic seizure in 2n, Ts and Tt mice.

PTZ dose (mg/kg)	PTZ-treated animals (N)			Clonic seizure incidence (N)			Clonic seizure incidence (%)		
	2n	Ts	Tt	2n	Ts	Tt	2n	Ts	Tt
60	18	18	18	4	1	5	22.2	5.6	27.8
70	14	18	14	9	10	7	64.3	55.6	50
75	31	45	30	22	34	25	71	75.6	83.3
80	19	30	12	12	21	11	63.2	70	91.7
90	18	17	16	16	14	16	88.9	82.4	100

The number of PTZ-treated mice, the number of convulsing mice and the percentage of convulsing mice are given for each genotype and PTZ dose.

**Table 2:** Effect of the dose of PTZ and of the genotype on the probability of seizure (logit scale).

Effect	Odds-ratio (95% Wald CI)	Wald Chisq test p-value
Dose (mg/kg)	1.14* (1.10–1.18)	<0.001
Mouse Type	-	0.12
2n	1	
Ts	0.92** (0.50–1.70)	
TT	1.79 (0.89–3.59)	

The odds-ratios (OR) are estimated from a logistic regression model. The dose of PTZ is the only statistically significant effect observed. Increasing the administered dose of one mg/kg leads to a significant increase of the probability of seizure. No significant odds-ratio was found when comparing Ts and Tt mice to 2n mice.

**Table 3:** Effects of the dose of *Cstb* and of the genotype on time of onset of tonico-clonic seizure.

	Clonic seizure latency and number of censored/N, mean* (standard error)						
Dose (mg/kg)	2n		Ts		Tt		Test of equality over genotypes§
60	14/18	708.8 (37.7)	17/18	470.0 (na)	13/18	941.0 (58.5)	0.23
70	5/14	608.6 (59.0)	8/18	547.2 (47.9)	7/14	807.8 (101.9)	0.82
75	9/31	587.4 (82.1)	11/45	468.3 (47.8)	5/30	541.4 (114.0)	0.21
80	7/19	342.3 (28.7)	9/30	625.8 (101.9)	1/12	453.1 (159.1)	0.13
90	2/18	338.9 (49.7)	3/17	497.8 (151.8)	0/16	437.0 (104.0)	0.92
Test of equality over doses§	0.0003		<0.0001		<0.0001		

The number of censored mice among the total number of mice, the mean latencies and their standard error estimations for each genotype group and dose of PTZ are presented.

§ Statistical comparison of genetic groups within each PTZ dose and comparison of doses within each genotype group were done using the log-rank test.

*Mean latencies and their standard errors estimations were restricted to the largest seizure time among non censored observations.

## Discussion

We report the generation and study of a mouse model bearing tandem duplication of the *Cstb* gene, leading to trisomy of the gene in *Cstb<tm1Yah>* heterozygous mice and to tetrasomy in homozygous mice. Presence of 3 copies of *Cstb* in Ts and of 4 copies in Tt mice results in respectively 2 and 3 folds over expression compared to control disomic expression. Two fold overexpression of *Cstb* in Ts mice is in accordance with reports of 2.15 fold change for *Cstb* expression in DS adult brains [18], 1.94 fold change in primary cultures of human fetal cells [35] and an average log ratio of 1.77 in astrocyte cell lines derived from fetal brains [36]. However, other studies, one on a lymphoblastoid cell line [37] and one on fetal hearts at 18–22 weeks of gestation [38], found that there was no significant difference in the levels of transcripts expression between the trisomic and diploid tissues analyzed. This indicates that compensatory mechanisms exist for the expression of *Cstb* in different tissues. It hence seems that, at least in the brain, adding one copy of the *Cstb* gene into the genome results in an about 2-fold over-expression of the transcripts in both human and mice. However, when looking at the protein level, the overexpression in Ts and Tt animals was lower than that observed at the mRNA level, indicating that some compensatory mechanism occurs at the translation level. The effect of the presence of a third gene copy on the expression of HSA21 genes in DS has been mostly studied at the transcript level following the development of high-throughput transcriptome analysis techniques. However, much less has been carried out on the proteome analysis and we could find no report about the level of Cystatin B in DS patients. Contrary to other T21 transgenic mouse models for single candidate genes [39]–[49] that often have a very high overexpression of the gene, our model seems to be more appropriated to study the effect of *Cstb* in T21. In addition, the role of *CSTB* in EPM1 characterized by an association of epilepsy, myoclonus and progressive neuronal deterioration [50] makes it a good candidate gene for the increased susceptibility of DS patients to epileptic seizures and the *Cstb<tm1Yah>* mouse provides a model to test this.

Although the susceptibility to epilepsy is higher in persons with DS than the general population, the mechanisms by which seizures are generated in DS have received little attention. Cognitive functions that are particularly affected in DS are spatial learning and memory [51]–[56], two functions that require the hippocampus and prefrontal cortex. Several studies suggested that impairment of these functions was the result of an alteration in the number of excitatory synapses [57]–[60]. This hypothesis was comforted by functional explorations of the hippocampus and the neocortex of Ts65Dn mice which presented an increased GABAergic inhibition [60]–[63] and by the beneficial effect of the use of GABA receptor antagonists on the memory performance of Ts65Dn mice [10], [11]. However, if epilepsy has been related to the alteration in the balance between excitation and inhibition, this change of balance is in the favor of an increased excitation and not an increased inhibition. Hence, additional mechanisms might underlie epileptic seizure in DS and finding the relationship between the epileptic anomalies and the presence of an extra Hsa21 remains essential not only to treat those symptoms, but also to be aware of all the possible side effects that might trigger the treatment of the cognitive impairment, especially with GABAergic antagonists such as PTZ.

We selected *CSTB* as a candidate Hsa21 gene for the increased susceptibility of DS persons to epileptic seizure because of its implication in EPM1, a type of myoclonic epilepsy characterized by stimulus-sensitive myoclonic seizures and slowly progressive cerebellar ataxia. 90% of EPM1 patients have an unstable dodecamer repeat expansion of at least 30 copies, located in the putative promoter of *CSTB* 175 bp upstream from the translation initiation codon, that leads to a drastic down regulation of *CSTB* gene expression [19]–[21], [50], [64]–[67]. Hence, the *CSTB* mRNA levels in patients homozygous for the expansion mutations was found to be less than 10% of that in the controls [67]. Whereas loss of function mutations in *CSTB* underlies the myoclonic epilepsy and progressive neurological deterioration observed in EPM1, the effect of increased *CSTB* expression in T21 is unknown and there is no evidence whether this gene is also responsible of the higher prevalence of epileptic seizures observed in the DS population. *CSTB* encodes cystatin B, a member of the cystatins or stefins family of protease inhibitors [68], [69], which main action is to inhibit the functions of cathepsins B, H, L and S, some lysosomal cysteine proteases [68]–[74]. Hence, cystatin B is thought to play a role in protecting against the proteases leaking from lysosomes, but little is known about its physiological functions and it probably also interacts with other cellular proteins. Di Giaimo and co-workers have reported in vitro interactions of CSTB with rat neurofilament light polypeptide, activated protein kinase C receptor, brain *β*-spectrin, a novel, myotubularin-related and a novel, unknown protein in cerebellar tissue, suggesting a role in cell growth and differentiation [75]. Discovery of neuronal apoptosis in the EPM1 mouse model deficient for cystatin B [25] and identification of increased expression of *Cstb* mRNA and protein in a rat kindling model of epilepsies [27] suggest a physiological role for this protein in the maintenance of normal neuronal structure. According to this, increased *Cstb* expression should result in a more protective role against epilepsy. Möller and collaborators (2001) however characterized epileptic features in DS patients with late onset myoclonic epilepsy and found generalized epileptiform discharges and EEG similar to those observed in EPM1 [22]. We cannot therefore exclude that over expression of *Cstb* akin to its deficiency, may produce epileptic phenotype via the perturbation of some molecular pathway balance. A convergent effect of both increase and decrease amounts of a same protein has already been described in the literature, with, for example, deregulation of Dyrk1A levels leading to motor and learning impairments in both heterozygous *Dyrk1A* mice and transgenic mice overexpressing *Dyrk1A* [1], [76], [77]. The finding that both over expression of wild-type or EPM1 mutants of cystatin B in neuroblastoma cells generates cytoplasmic aggregates [30] has suggested that cystatin B in vivo has a polymeric structure sensitive to the redox environment. Knowing that evidence of oxidative stress was reported in individuals with DS, it is not inappropriate to postulate that increased *Cstb* expression might play a role in increased epileptic susceptibility observed in DS. EPM1 onset has been related to latent hyperexitability and, upon discovery of reduced density of GABA-immunoreactive cells in the hippocampus of *Cstb*-deficient mice, scientists have postulated that loss of GABAergic inhibition plays a role in this phenomenon [26]. We therefore analysed Ts and Tt mice for both EPM1-like phenotype and tested the effect of increased expression of *Cstb* on the susceptibility or resistance to PTZ-induced clonic seizures.

Observation and analysis of Ts and Tt mice for locomotion activity in an open-field did not reveal any gross lack of coordination of muscle movements such as wide-based gait or falling upon hindlimb rearing that are characteristic of ataxia. Whereas a slight decreased in performance was found for Tt males in their ability to walk on the rotating rod at session 6, this difference was not found again in the later sessions, and no significant locomotor deficit could be measured in transgenic animals. Morphological and histological analysis of the cerebellum did not reveal any atrophy or severe decrease of the density of the granule cell layer, the neuropathological hallmark of the *Cstb*-deficient mice [25]. EEG analysis enables to visualize events such as interictal discharges (spikes) and the recently discovered high frequency activities (fast ripples) in epileptic patients or animal models even in the absence of a seizure. EEG recorded in the 2n, Ts and Tt mice showed a strictly normal activity, indicating that these animals were not epileptic. Looking for the implication of *Cstb* in susceptibility to PTZ-induced seizure, we subjected 2n Ts and Tt animals to increased dose of PTZ. Whereas there was an effect of the increasing dose of PTZ on the number of convulsing mice and the latency time between the injection and convulsion, no effect of the genotype could be observed. Our analysis did not reveal any increase in susceptibility or resistance to PTZ of the Ts and Tt mice compared to 2n and increased expression of *Cstb* does not alter susceptibility to tonico-clonic induced seizure triggered by PTZ. Presence of 3 copies of the *CSTB* gene is probably not involved in susceptibility to epileptic seizure in the DS population. However, we cannot rule out an involvement of this gene in seizure susceptibility, not via an increased of dosage, but due to the presence of more than one allele of the same deleterious allelic variant, which then becomes sufficient to overcome the buffering of a normal allele, a mechanism that has already been suggested for the pathologic contribution of trisomic genes in DS. Indeed, whereas EPM1 is rare in western Europe, it was found to be common in north Africa where about 60% of the patients share the same haplotype, thus establishing a founder effect in this population [78]. Furthermore, in addition to down-regulation of gene expression found in 90% of the EPM1 cases, five coding-region mutants have also been found, usually heterozygous with the repeat expansion [67]. Among these mutants, mutants R68X and G4R were more especially prone to form aggregates in cells [79]. This allelic variant affect would explain the penetrance of the epileptic phenotype in DS. In this point of view, it would be of interest to analyse the genetic background that is known to influence the presence or severity of myoclonic seizures in *Cstb−/−* mutant mice [25].

Increased susceptibility to epilepsy in DS patients is surprising regarding the finding that they have been shown to have excess GABA-mediated inhibition [80]. Nervertheless other mechanisms might underlie epileptic susceptibility in DS and additional Hsa21 genes could influence seizure-susceptibility. Searching for Hsa21 mouse orthologous genes implicated in seizure according to mammalian phenotype ontology using the Mouse Genome Informatics (MGI) group (www.informatics.jax.org), five genes in addition to *Cstb* were listed that, when mutated, are associated with a variety of seizure related phenotypes. Among those genes, *Kcnj6* (*Girk2*) codes for a ion channel subunit, ion channels representing 66% of the currently known Mendelian human epilepsy gene, and a point mutation in this gene causes ataxia, tremor and tonico-clonic seizures in the weaver mouse [81]. However, studies in human have so far failed to demonstrate an association between KCNJ6 and epilepsy [82], [83]. *Dscam*, a member of the immunoglobulin (Ig) superfamily, has been implicated in cell migration and sorting, axon guidance, formation of neural connections and synaptic plasticity [84], [85]. *Synj1* codes for a nerve terminal protein that appears to be involved in synaptic vesicle recycling [86] and its presence in three copies in the Ts65Dn model results in altered phosphatidylinositol-4,5-bisphosphate metabolism in the brain [87]. The transcription factor encoding gene *Olig2* is implicated in glial development [88] and neuronal repair after brain injury [89]. These first four genes are found on the trisomic region present in the Ts65Dn model that served to test chronic low-dose PTZ treatment of T21 memory deficits. The last gene, however, is present on Mmu10: *Adarb1* is a gene coding for a RNA-editing enzyme widely expressed in brain and its inactivation in mouse leads to seizure susceptibility and could hence have an influence on susceptibility to PTZ treatment [90]. Finally, SOD1, a major cytoplasmic antioxidant enzyme converting superoxide radicals to molecular oxygen and hydrogen peroxide and whose gene is present on Hsa21 and Mmu17, was recently identified as a possible epileptic biomarker in a proteomic analysis of cerebrospinal fluid (CSF) from patients with temporal lobe epilepsy (TLE) [91]. Hence, other gene candidates remain to be tested and there are still concerns over what the long-term effects of chronic pro-epileptic drug such as PTZ might be in a population at higher risk of epileptic seizure such as DS people.

## Materials and Methods

### Generating a tandem duplication of the *Cstb* gene on MMU10

A tandem duplication of *Cstb* was obtained during the insertion by homologous recombination of a targeting vector for *Cstb* isolated from the *5′Hprt* library [32] in HM-1 embryonic stem (ES) cells [33]. The region of homology in the insertion vector contains the three alleles of *Cstb* and the insertion of the vector leads to the formation of two entire *Cstb* genes separated by the vector backbone (Fig. 1). Recombinant ES cell clones were selected against neo and verified by Southern blot analysis using two external probes. They were then injected into C57BL/6J (B6) blastocysts to generate chimera. These animals were crossed with B6 mice to obtain the corresponding mouse line, named *Cstb<tm1Yah>*. After establishment of the line on the B6 background (backcross level higher than N7), heterozygous mice (one chromosome containing the tandem duplication and one wild-type chromosome) were intercrossed to give disomic (2n; two wild-type chromosomes), trisomic (Ts; heterozygous for the chromosome containing the tandem duplication) and tetrasomic (Tt; homozygous for the chromosome containing the tandem duplication) mice for *Cstb*.

### Mouse genotyping

Mice were genotyped by Southern blot analysis in standard conditions. Briefly, 10 µg of genomic tail or ES cell DNA extracts were digested with the *Bst*XI enzyme and separated by electrophoresis through 0.8% agarose gel. The digested DNA was transferred to Hybond nylon membrane (GE Healthcare, Chalfont St Giles, UK) and hybridized with a specific DIG-labeling probe (probe B in Fig. 1) (Roche, Mannheim, Germany). Autoradiography was performed using Kodak Biomax XAR-Films (Kodak, Chalon-sur-Saône, France).

### RNA extraction and quantitative RT-PCR analysis

Total RNA was extracted from the liver and from the brain of 2n, Ts and Tt mice using the RNAeasy^R^ mini-kit (Qiagen) and RNA integrity was checked using the Agilent 2100 bioanalyzer. cDNA synthesis was performed using the Absolute™ 2-step QRT-PCR SYBR Green Kit (ABgene, Epsom, UK). The primer pairs used for QPCR amplification of *Cstb* and of the selected normalization gene (*Actb*) are the ones designed by Lyle et al. (2004) [92]. Primers for *Cstb* are in exon 2 and exon 3 of the gene. HPLC purified FAM-TAMRA-labeled (*Cstb*) and HEX-TAMRA-labeled (*Actb*) double dye Taqman probes were obtained from Eurogentech. Efficiency of the Taqman assay was checked using a cDNA dilution series from extracts of brain samples [93]. The QPCR was performed with 15 ng of cDNA and 200 nM of each primer in a 15 µl final reaction that contained primers for both the tested (*Cstb*) and normalization gene (*Actb*) in a Stratagene Mx4000 with a standard amplification procedure. Reactions were made in triplicate and the mean Ct used for measurement of the transcript levels that were normalized against transcript levels of *Actb* in order to correct the variations of the amount of source RNA in the starting material. For each tissue, the QPCR analysis was repeated three times (three plate runs) to check for reproducibility of the results.

### SDS-PAGE and Western blot

Ten microgram of total protein from brain extracts were electrophoretically separated in SDS-polyacrylamide gels (17%) and transferred to nitrocellulose membrane. Non-specific binding sites were blocked with 5% skim milk powder in Tween Tris buffer saline for 1 h at room temperature. Immunostaining was carried out with a rabbit polyclonal anti-Stefin B (ab53725 from AbCam) and a mouse monoclonal anti-β-tubulin, followed by secondary anti-rabbit IgG and anti-mouse IgG conjugated with horseradish peroxidase. The immunoreactions were visualized by ECL chemoluminescence (Amerham Biosciences) and exposure to ECL Hyperfilm (GE healthcare). Semi quantitative analysis was performed using the ImageJ software (W. Rasband, NIH; http://rsb.info.nih.gov/ij/).

### Statistics

All quantitative results were presented as mean±s.e.m. (standard error of the mean). For statistical analysis, levels of mRNA and protein transcripts in Ts and Tt animals were compared to levels in 2n mice by performing either the parametric Fischer-Student *t*-test when applicable (when normality and equal variance tests passed) or the non parametric Wilcoxon Mann-Withney's *U*-test via the Statgraphics software (Centurion XV, Sigma plus, Levallois Perret).

### Spontaneous locomotor activity and rotarod

Animals were bred under SPF conditions and were treated in compliance with animal welfare policies from the French Ministry of Agriculture (law 87 848). YH, as the principal investigator in this study, was granted the accreditation 45-31 to perform the reported experiments.

Fifteen diploid (2n), 10 Ts and 8 Tt mice (4–6 months) were used in these tests. Spontaneous locomotor activity was measured between 8.00 am and 13.00 pm. Each animal was individually placed in an open-field (Acti-track, Panlab; 43×43×35 cm, 10 Lux) for a 30 min session. The locomotor activity was evaluated by measures of distance travelled and number of rears [94]. Rotarod testing was conducted between 8.00 am and 16.00 pm in an accelerating rotarod (TSE). Animals were subjected to nine 10-min sessions. Mice were placed on the rod, rotating at an initial speed of 1 rpm; the speed was progressively increased from 1 rpm to 5 rpm on the first five sessions (pretraining) and from 1 to 20 rpm on the following sessions. The mice were placed again on the rod after two falls and were removed from the apparatus after the third fall. The latency of the third fall was recorded (a score of 600 sec was given when the mouse fell less than three times). Statistical analysis was done comparing each transgenic group versus the disomic (2n) group using ANOVA followed by Student t-tests in case of a significant Fisher test (*p*<0.05).

### Histological analysis

Mice aged over 6 month (2 of each genotype) were deeply anesthetized with pentobarbital and perfused intra-cardially with 30 ml of phosphate-buffered saline (PBS), followed by perfusion with 30 ml 4% paraformaldehyde in phosphate buffer. The brain was dissected and post-fixed overnight in 4% PFA at 4°C, dehydrated, embedded in paraffin and sectioned at 5 µm. Sections were dewaxed, rehydrated and stained with hematoxylin and eosin.

### Electrophysiological evaluation

Presence of epileptic events was checked in freely moving animals via electrophysiological recordings of 3 mice from each genotype (2n, Ts and Tt). EEG recording was done by implantation of skull surface electrodes two weeks before the start of recording. Animals were put under general chloral hydrate anesthesia (400 mg/kg) and midline scalp incision was made to expose the skull. Five surface screw unipolar recording electrodes were implanted, four bilaterally 2.5 mm from midline in the frontal and parietal cortex, and the last one above the cerebellum as reference. Electrodes were sealed to the dental acrylic. Animals were allowed to recover for 15 days before experimental analysis. EEG activity was recorded in both hemispheres with a numerical acquisition system (Coherence, Deltamed) to detect any hypothetical synchronized EEG activity. Animals were connected to the recording system and tested within a Faraday cage. After a 15 minute habituation delay, mice were recorded for 30 min for the two first trios (one mouse from each genotype) and overnight for the last trio.

### Testing *Cstb* trisomic and tetrasomic mice to PTZ-induced seizures

To test if the presence of additional copies of *Cstb* increases the susceptibility of the mice to convulsant agents, seizures were induced in 2n, Ts and Tt mice by administration of PTZ at doses of 60, 70, 75, 80 and 90 mg/kg, with different groups of animals being injected for each dose. Hence, each mouse was used only once. As this represents a total of 318 animals that could not be generated at the same time, 10 groups of animals were produced, each one containing the 3 different genotypes, and were tested between 10 and 14 weeks of age. All the animals in one group were tested the same day for one dose of PTZ. All experiments were conducted in the same conditions between 12:00 am and 2:00 pm. Number of animals tested for each genotype and PTZ doses are detailed in Table 1. The mice were kept in colony cages with free access to food and tap water, under standard housing conditions. Mice were weighted and the individual body weight used for dose volume calculation of the PTZ. The PTZ was injected subcutaneously. Following the injection of PTZ, mice were placed separately into transparent Plexiglas cages and observed for 30 min for the occurrence of tonico-clonic seizures. The tonico-clonic seizure was defined as clonus of the whole body lasting over 3 sec, with an accompanying loss of righting reflex. The number of animals convulsing out of the total number of mice tested was scored for each genotype. For animals that were convulsing, the latency time between the injection and the first tonico-clonic seizure was recorded for each mouse. Because males and females did not differ in seizure susceptibility, the data consist of measurements obtained from both groups that were represented by an approximately equal number of animals. For ethical reason, the few animals that have entered in a status epilepticus after that the tonico-clonic seizure has stopped were sacrified after 1 min. The study data were summarized and tabulated by genotype group for the percentage of convulsing mice (Table 1). For each genotype, the dose-response curve (number of convulsing mice/total number of injected animals according to the PTZ dose) was plotted and fitted by a logit model (Fig. 5a). The probability of seizure based on the genotype of the mouse (categorical predictor), the PTZ dose (continuous predictor), and their interaction was then calculated using a multivariate logistic regression model. Odds-ratios (OR) for the PTZ dose and for the increased *Cstb* gene dosage (2n<Ts<Tt) was calculated as followed:

* OR for increasing the dose of one unit : = 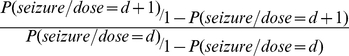



** OR for mouse Ts compared to mouse 2n: = 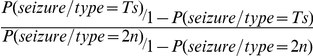



The effects of the genotype and of the dose of PTZ on the latency of the PTZ-induced seizure were evaluated by a stratified comparison of the latency times of the 3 genetic groups using log-rank tests within each dose of PTZ, because the plot of the survival curves (Fig. 5c) of the 3 genotype groups showed that the proportional hazard assumption was violated, and suggested that a Cox model analysis was not applicable. For this analysis, latencies were right censored at 1800 sec (30 min observation).
